# Neurogenesis defects in iPSC-derived midbrain organoids of early-onset Parkinson's disease with 22q11.2 deletion syndrome

**DOI:** 10.3389/fncel.2026.1906023

**Published:** 2026-07-31

**Authors:** Syugo Ueki, Toshiya Kimura, Rie Tohge, Atsushi Tamada, Mitsuaki Oki, Yusuke Yakushiji, Haruhisa Inoue, Satoshi Kaneko, Keiko Muguruma

**Affiliations:** 1 Department of Neurology, Kansai Medical University, Hirakata, Japan; 2 Department of iPS Cell Applied Medicine, Graduate School of Medicine, Kansai Medical University, Hirakata, Osaka, Japan; 3 iPSC-based Drug Discovery and Development Team, RIKEN BioResource Research Center (BRC), Kyoto, Japan; 4 Center for iPS Cell Research and Application (CiRA), Kyoto University, Kyoto, Japan; 5 Medical-Risk Avoidance Based on iPS Cells Team, RIKEN Center for Advanced Intelligence Project (AIP), Kyoto, Japan; 6 Department Neurology, Medical Research Institute Kitano Hospital, Osaka, Japan

**Keywords:** 22q11.2 deletion syndrome, dopaminergic neuron, floor plate, induced pluripotent stem cells, midbrain organoid, Parkinson's disease

## Abstract

Sporadic Parkinson's disease (PD) is typically a late-onset disorder caused by a combination of genetics, environment, and aging, manifesting when the loss of midbrain dopaminergic neurons exceeds a critical threshold, usually after the age of 50. Conversely, early-onset PD, as observed in cases linked to *parkin (PRKN)* gene mutations, suggests mechanisms involving either accelerated postnatal neuron loss or an insufficient number of neurons at birth. Patients with the 22q11.2 deletion syndrome (DS) have a significantly higher prevalence of early-onset PD. The absence of known genes associated with hereditary PD in the deleted region suggests the involvement of novel, non-traditional risk factors. This could potentially implicate the neurodevelopmental origin of dopaminergic neurons arising from the floor plate. To investigate this hypothesis, we generated midbrain organoids from induced pluripotent stem cells derived from a patient with 22q11.2 DS. The organoids recapitulated key aspects of *in vivo* neurogenesis, revealing enhanced differentiation of dopaminergic neurons in 22q11.2DS- and *PRKN*-derived organoids compared to controls on days 28 and 56 of culture. These findings suggest that, in early-onset PD patients with 22q11.2 DS or *PRKN* mutation, enhanced neurogenesis could result in reduced number of dopaminergic neurons during early development. The organoids of early-onset PD demonstrated that progenitors undergo enhanced differentiation at an early stage. This suggests that the atypical developmental process could reduce progenitors before there are enough mature dopaminergic neurons. This in turn indicates that the onset of PD may occur as early as the embryonic stage.

## 1 Introduction

Sporadic Parkinson's disease (PD) is thought to result from a combination of genetic background, environmental factors, and aging processes. Motor symptoms such as tremor and bradykinesia appear when the number of midbrain dopaminergic neurons decreases and the threshold is exceeded. The onset of motor symptoms usually occurs between the ages of 50 and 65, at which point the number of dopaminergic neurons in the substantia nigra is reduced to less than half of normal levels. In contrast, PD with Parkin RING-between-RING E3 ubiquitin-protein ligase (*PRKN*) gene mutations and some other cases of hereditary PD are known to occur before the age of 40. Two possible mechanisms have been proposed for the onset of the disease at a young age: an accelerated loss of dopamine neurons after birth or a low number of dopamine neurons at birth (Laperle et al., 2020); however, the mechanisms have not yet been clarified. Clarifying the relationship between the onset of PD and qualitative and/or quantitative abnormalities in dopamine neurons is likely to provide clues for therapeutic strategies to prevent the onset and progression of the disease; therefore, there is a strong need to identify the underlying cause.

22q11.2 deletion syndrome (DS) is a rare disease occurring in approximately 1 in 4,000 to 5,000 people, with approximately 90% of cases involving a deletion of a 3 Mb region of chromosome 22q11.2. The main clinical symptoms are congenital heart disease, as well as developmental abnormalities such as impaired formation of midline structures, including the pharyngeal arches and thymus, and cleft palate and soft palate incompetence. Furthermore, more than 60% of cases are accompanied by neuropsychiatric disorders such as developmental or behavioral disorders, attention deficit hyperactivity disorder, and schizophrenia (Boot et al., 2019). Recently, 2.5% of patients with 22q11.2 DS develop early-onset PD (Butcher et al., 2013), which is significantly higher than the prevalence of PD in the general population (150–200 per 100,000). In 22q11.2 DS, there are approximately 60 genes in the deleted region, but none of them are associated with hereditary PD (Butcher et al., 2017). This suggests the existence of risk factors that are different from those involving direct control of disease onset by genetic mutations.

Midbrain dopamine neurons originate from the floor plate located in the ventral midline of the neural tube (Ono et al., 2007), suggesting that the onset of PD might be attributable to developmental abnormalities in dopaminergic neurons in 22q11.2 DS. Based on this hypothesis, we investigated the pathogenesis of PD associated with 22q11.2 DS by focusing on neurogenesis and analyzing midbrain organoids generated from patient-derived induced pluripotent stem cells (iPSCs). Neural organoids are useful for exploring human neurogenesis (Muguruma et al., 2015; Xiang et al., 2017). Organoid technology has contributed to our understanding of neural development and disease pathogenesis (Tamada and Muguruma, 2024).

We reported the first case in Japan of 22q11.2 DS that developed early-onset PD (Oki et al., 2016) and further analyzed the clinical characteristics in an international collaborative study (Boot et al., 2018). In this study, we aimed to elucidate the pathogenic mechanisms of PD associated with 22q11.2 DS. To this end, we generated midbrain organoids from patient-derived i PSCs and analyzed the development of dopaminergic neurons. In addition, iPSCs from healthy volunteers, unaffected family members, and patients with early-onset PD carrying *PRKN* mutations were included as comparative controls. By examining dopaminergic neuronal development across these distinct genetic backgrounds, we sought to clarify developmental abnormalities and provide a cross-sectional understanding of the pathogenic mechanisms underlying early-onset PD.

## 2 Materials and methods

### 2.1 iPSC culture and organoid differentiation

The use of human iPSCs (hi PSCs) was approved by the Ethics Committees of RIKEN Kobe Branch (KOBE-IRB-16-34, January 6, 2017), RIKEN Bio Resource Research Center (BRC) (RIKEN-T-2025-005, July 25, 2025) and Kansai Medical University (2018024, June 4, 2024). For the generation of hi PSCs from a patient with 22q11.2 DS and a blood relative, *OTX2, SOX2, KLF4, L-MYC, LIN28, EBNA1*, and p53 carboxy-terminal dominant-negative fragments were transduced into peripheral blood mononuclear cells using episomal vectors as previous described (Okita et al., 2011, 2013). All participants provided written informed consent. The hi PSCs, HPS4264 and HPS4265, were independent clones derived from the same donor and obtained from the RIKEN BRC as an early-onset PD line harboring the homozygous c.1341G>A (p.W447Ter) variant in the PRKN gene. The healthy participant-derived hiPSC line, HC6#10, was used as a control (Ishida et al., 2016; Kamei et al., 2023; Kume et al., 2023). The information of hi PSCs used in this study is shown in Supplementary Table 1. All hi PSC lines used in this study were maintained and analyzed between passages P13 and P45. All cell lines were confirmed to be free of mycoplasma contamination at establishment using the TaKaRa PCR Mycoplasma Detection Set (#6601).

To maintain the cells, hi PSCs were cultured on laminin (iMatrix-511; Nippi) in StemFit AK02N (Ajinomoto) at 37°C in a 5% CO_2_ incubator. Cells were passaged every 7 days. For passaging, hiPSC colonies were treated with 0.5 × TrypLE Express Enzyme (Thermo Fisher Scientific) and dissociated into single cells by gentle pipetting. Dissociated hi PSCs were re suspended in Stem Fit AK02N and seeded at a density of 1,300 cells/cm^2^ onto iMatrix-511–coated dishes in StemFit AK02N supplemented with 10 μM Y-27632 ROCK inhibitor (Tocris). The medium was replaced the following day with Stem Fit AK02N without Y-27632 and subsequently changed every other day. Midbrain organoids were generated by neural differentiation of hi PSCs, as previously described (Jo et al., 2016; Smits et al., 2019) with slight modifications. Briefly, dissociated hi PSCs were rapidly re-aggregated in a 96-well V-bottom low-cell adhesion culture plate (Prime Surface MS-9096V, Sumitomo Bakelite) at a density of 10,000 cells per well in 75 μl of induction medium containing 80% Knock Out™ DMEM, 20% Knock Out Serum Replacement, 1% non-essential amino acids solution, 1% penicillin/streptomycin (P/S), 1% GlutaMAX (all reagents from Thermo Fisher Scientific), 100 μM 2-mercaptoethanol (2-ME) (Sigma-Aldrich), freshly supplemented with 10 μM SB431542 (Tocris), 250 nM LDN-193189 (Tocris), 0.8 μM CHIR99021 (Stem gent), 0.5 μM SAG (Enzo), and 5 μM Y-27632 in 5% CO_2_ incubator. The day of initiation of the serum-free floating culture of embryoid body-like aggregates with quick reaggregation (SFEBq) was defined as day 0.

On day 2, an equal volume of maturation medium containing 50% DMEM-F12, 50% Neurobasal medium, 1x GlutaMAX, 1x N-2 supplement, 1x B-27 supplement (minus vitamin A), 1x P/S, 100 μM 2-ME, freshly supplemented with 10 μM SB431542, 250 nM LDN-193189, 0.8 μM CHIR99021, and 0.5 μM SAG was added. On day 4, half-volume replacement with maturation medium freshly supplemented with 10 μM SB431542, 250 nM LDN-193189, 0.8 μM CHIR99021, 0.5 μM SAG, 200 μM ascorbic acid (AA), and 100 ng/ml recombinant FGF8b (PeproTech) was performed. On day 7, half-volume was replaced with maturation medium freshly supplemented with 0.8 μM CHIR99021, 0.5 μM SAG, 200 μM ascorbic acid (AA), and 100 ng/ml FGF8b, resulting in a reduction of the SB and LDN concentration to half of the original values. On day 10, two-thirds of the volume was replaced with maturation medium freshly supplemented with 0.8 μM CHIR99021, 0.5 μM SAG, 200 μM AA, and 100 ng/ml FGF8b. On day 14, two-thirds of the volume was replaced with maturation medium freshly supplemented with 1% Matrigel Growth Factor Reduced (Corning), 10 ng/ml BDNF (PeproTech), 10 ng/ml GDNF (PeproTech), 200 μM AA, and 500 μM db-cAMP (Sigma-Aldrich). The medium was changed every 3–4 days thereafter. For quantitative analyses, multiple independent differentiation experiments were performed for each iPSC clone. At day 28, FOXA2/LMX1A-positive cells were quantified in 197 organoids, and TH-positive cells were quantified in 283 organoids. At day 56, TH-positive cells were quantified in 161 organoids. Detailed information regarding the number of independent differentiation batches and analyzed organoids for each iPSC clone is provided in Supplementary Table 3.

### 2.2 Immunohistochemistry

Organoids were fixed in 4% paraformaldehyde in PBS for 20 min, washed in PBS, and immersed in 30% sucrose in PBS overnight at 4°C. Organoids were then embedded and frozen in Tissue-Tek Optimal Cutting Temperature (O.C.T., Sakura), cryosectioned at a 14-μm thickness. Immunohistochemistry was performed as described previously (Muguruma et al., 2015; Kamei et al., 2023) with the primary and secondary antibodies described in Supplementary Table 2. Nuclear counterstaining was performed with DAPI.

### 2.3 Microscopic imaging

Images were acquired with confocal microscopes (LSM700, LSM710, Zeiss) equipped with Zen software. All images were processed using Adobe Photoshop CC24. The number of cells was counted using HALO (version 3.6.4134, Indica labs). For quantitative analysis, one representative section per organoid was analyzed. Among 3–5 sections obtained from each organoid and mounted on a single slide, the section with the largest cross-sectional area was selected according to an objective structural criterion without additional pre-screening or subjective exclusion. First, a composite image was binarized to detect the surface of an organoid using Image J. Analysis was restricted to the region less than 200 μm deep from the surface to minimize the contamination of dying cells, which were usually abundant at the deeper region. In cases in which the organoid lacks a part due to ejection of cells during the culture, a smooth arc was drawn between the gap of the surface outline so as to fill in the missing part. Cell nucleus was defined as profiles mildly stained with DAPI and of an oval shape and automatically recognized with HALO. Tyrosine hydroxylase (TH)-positive cells were defined as profiles exhibiting fluorescence staining within 1 μm of the nuclei identified using DAPI signals, and automatic quantification was performed with HALO. Image quantification was performed using HALO with predefined analysis parameters and fixed thresholds to minimize operator-dependent bias.

### 2.4 Karyotype and fluorescence *in situ* hybridization

KaryoMAX (Thermo Fisher Scientific) was added to hiPSCs maintained under standard culture conditions, and the cells were incubated for 6 h at 37°C in a CO_2_ incubator. hiPSCs were treated with 0.068 M KCl for 10 min and fixed with Carnoy fixative. Karyotyping with Giemsa staining was performed. Fluorescence *in situ* hybridization (FISH) was performed using SpectrumOrange-labeled TUPLE1 (HIRA) and SpectrumGreen-labeled arylsulfatase A (ARSA) probes.

### 2.5 Sequencing of PRKN-mutant hiPSCs

Genomic DNA from *PRKN*-mutant hiPSCs was extracted using Proteinase K (without R Nase A). Extracted DNA was amplified using Prime STAR GXL DNA polymerase (Takara Bio), purified and sequencing was carried out with primers PRKN_genome_ex12_Fw3 (AGAGCTGCCCTATTGTGCTT) and PRKN_genome_ex12_Rv3 (GCTCTGCTGTCTTGTGTGGA). Sanger sequencing was performed on the PCR product using the reverse primer PRKN_genome_ex12_Rv2 5′-CTTGAAGAGTGTGTGTGCGC3′).

### 2.6 Statistical analysis

All data, shown as mean ± S.E.M. obtained from independent experiments (n≥3), were statistically analyzed with PRISM (ver. 10.6.1, GraphPad). When homogeneity of variance was confirmed, a one-way ANOVA was performed, followed by Dunnett's *post hoc* multiple comparison test to compare each group with HC6. When homogeneity of variance was violated, the Kruskal–Wallis test was conducted, followed by Dunn's *post hoc* multiple comparison test to compare each group with HC6. *p* < 0.05 was considered statistically significant.

## 3 Results

### 3.1 Generation of early-onset PD patient iPSCs

**Figure 1:**
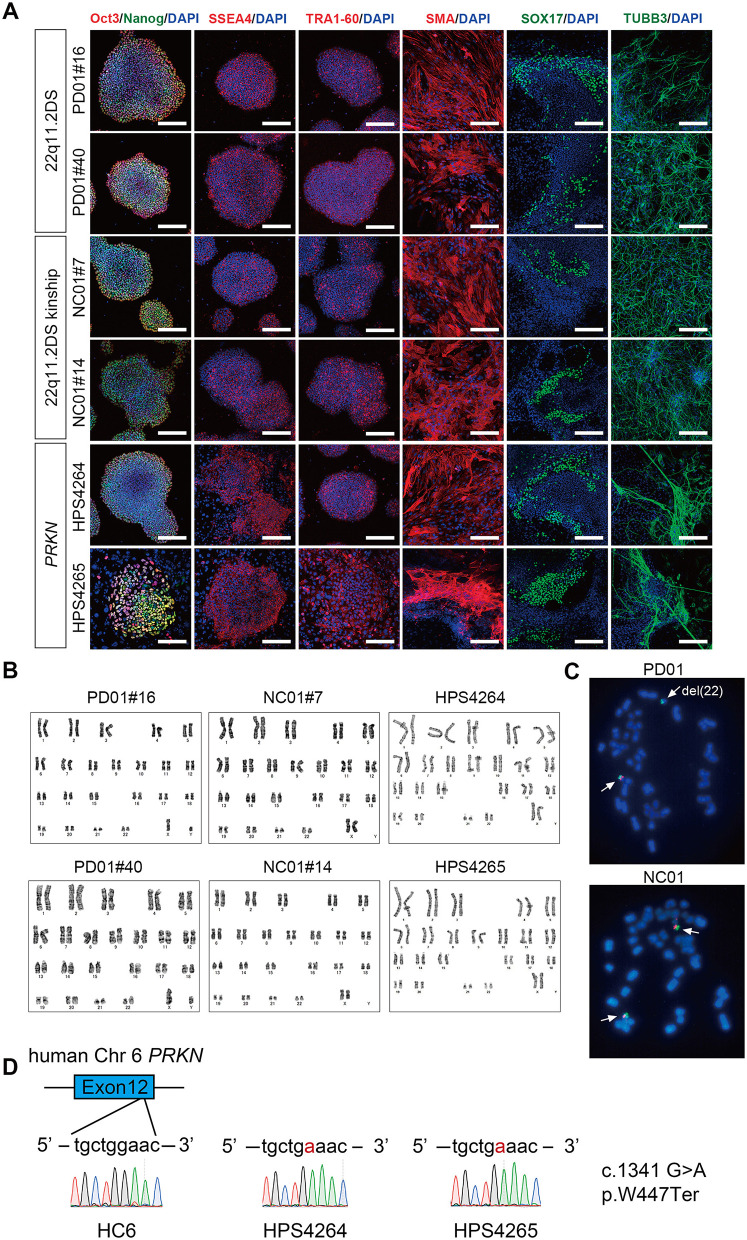
Generation and Characterization of iPSCs. **(A)** Morphology and pluripotency marker expression pattern of iPSC lines derived from a patient with 22q11.2 deletion syndrome (DS) (PD01#16 and PD01#40), their blood relative (NC01#7 and NC01#14), and a patient with a genetic mutation in the PRKN gene (HPS4264 and HPS4265). iPSCs expressed pluripotent stem cell markers including NANOG, OCT3/4, SSEA4, and TRA1-60. The three-germ layer differentiation capacity of the iPSCs was shown by immunohistochemical staining of SMA (mesoderm), SOX17 (endoderm), and TUBB3 (ectoderm). Scale bars, 200 μm. **(B)** All iPSC lines exhibited normal karyotypes. **(C)** FISH analysis demonstrated genetic defects in the 22q11.2 DS iPSCs (PD01) but not in their blood relative (NC01). Arrows indicate Spectrum Orange-labeled TUPLE1 (HIRA) and Spectrum Green-labeled arylsulfatase A (ARSA). **(D)** Representative genomic DNA sequences of exon 12 of the PRKN gene in healthy control (HC6) and patient with PD-derived iPSCs (HPS4264 and HPS4265). Sanger sequencing confirmed that PD iPSC clones carried the c.1341G>A (p.W447Ter) variant in the PRKN gene.

In the current study, we generated iPSCs from a PD patient with 22q11.2 DS (Oki et al., 2016) (PD01#16 and PD01#40) and an unaffected blood relative (NC01#7 and NC01#14) (Oki et al., 2016). iPSCs derived from a healthy control participant (HC6#10) (Ishida et al., 2016; Kamei et al., 2023; Kume et al., 2023) and an early-onset PD patient harboring *PRKN* mutations (HPS4264 and HPS4265) were used as controls. The pluripotency and self-renewal capacity of the iPSCs were confirmed (Figure 1A). G-banding karyotype analysis demonstrated no numerical or structural chromosomal abnormalities in any of the cell lines (Figure 1B). Fluorescence *in situ* hybridization (FISH) revealed heterozygous deletions at the 22q11.2 locus in PD01, but not in NC01 (Figure 1C). Sanger sequencing identified the c.1341G>A (p.W447Ter) mutation within exon of the *PRKN* gene in HPS4264 and HPS4265, whereas no such mutation was detected in HC6#10 (Figure 1D).

The HC6 iPSC line has been used in various studies of neurological disorders. As this iPSC line has been reported to differentiate into cerebellar organoids (Kamei et al., 2023), Purkinje cells (Ishida et al., 2016), spinal motor neurons (Kume et al., 2023), anterior pituitary cells and hypothalamic cells (Matsumoto et al., 2020), it was considered suitable for use in the differentiation of dopaminergic cells and midbrain organoids.

### 3.2 Characterization of neuroepithelial structures and the floor plate in organoids

**Figure 2:**
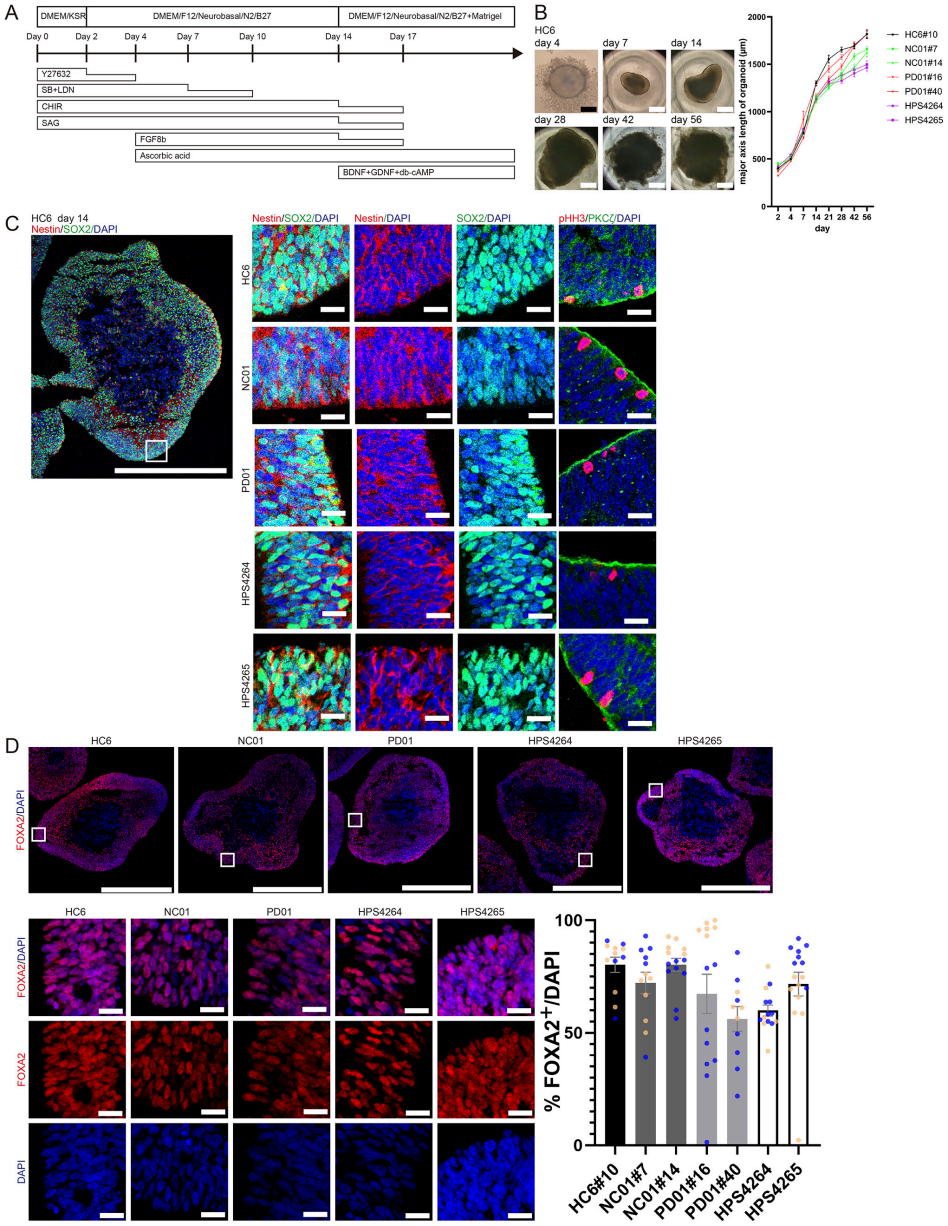
Differentiation and characterization of iPSC-derived midbrain organoids. **(A)** Schematic illustration of the differentiation protocol for midbrain organoids. **(B)** Representative bright-field images of organoids derived from the HC6 line at different time points and their corresponding size measurements. Scale bars, 200 μm (black), 500 μm (white). **(C)** Expression of neural stem cell markers SOX2 (green) and Nestin (red) in organoids on day 14. A representative image of an HC6-derived organoid is shown on the left. Scale bar, 500 μm. The images on the right are magnified views of the neuroepithelium within the organoid. The Nestin/SOX2/DAPI image in the top row is a magnification view of the HC6-derived organoid inset shown in the figure on the left. PKCζ was expressed at the organoid surface, and pHH3-positive nuclei were aligned along PKCζ expression. Scale bars, 20 μm. **(D)** Immunostaining for FOXA2 in iPSC-derived organoids cultured for 14 days. The insets in the top row were magnified in the below images. The scale bars represent 500 μm for low-magnification images and 20 μm for magnified images. The percentage of FOXA2^+^ cells on day 14 from 12–16 organoids across two independent batches for each iPSC line is presented. Each dot represents an individual organoid, and colors indicate independent differentiation batches within each clone.

Midbrain organoids were generated from control and PD iPSCs according to previously described protocols (Jo et al., 2016; Smits et al., 2019) with slight modifications (Figure 2A). Neural induction was initiated by dual SMAD inhibition with SB431542 (a TGFβ/Activin pathway inhibitor) and LDN-193189 (a BMP pathway inhibitor). Anteroposterior patterning toward the midbrain was achieved using CHIR99021 (a GSK-3 inhibitor) and FGF8, whereas dorsoventral patterning toward a ventral fate was induced with SAG. Neuronal growth was promoted by supplementation with ascorbic acid (AA), BDNF, GDNF, and db-cAMP. To minimize variability between experiments, midbrain organoids were differentiated from all cell lines simultaneously. The healthy control (HC6#10) neural organoids demonstrated a marked increase in size, growing to a diameter of more than 1 mm by day 14 in culture and continuing to increase in size (see black line in Figure 2B). Minor difference was observed in size between organoids derived from iPSCs with the 22q.11.2 deletion (PD01#16 and PD01#40), their blood relative (NC01#7 and NC01#14), and those harboring *PRKN* mutations (HPS4264 and HPS4265), compared with organoids from the healthy control (Figure 2B).

On day 14 of culture, neural stem cell markers SOX2 and nestin (NES) were expressed in organoids derived from PD iPSCs and in control organoids (Figure 2C). Continuous SOX2^+^/NES^+^ neuroepithelial structures had formed at the periphery of the organoids with apical-out and basal-in polarity. The organoids reproducibly formed PKCζ at the apical surface, and phosphorylated histone H3 (pHH3), a marker of the G2–M phase of the cell cycle, was detected adjacent to the apical surface (high-magnification view in Figure 2C).

Floor plate cells are morphologically specialized organizer cells located along the ventral midline of the developing neural tube caudal to the diencephalon (Placzek and Briscoe, 2005). Floor plate cells acquire neural progenitor characteristics and generate midbrain dopaminergic neurons. Forkhead box A2 *(FOXA2)* is expressed in the floor plate and functions as a transcription factor for midbrain dopaminergic neuron development (Ono et al., 2007). In organoids cultured for 14 days, FOXA2^+^ cells were highly expressed in the neuroepithelium (Figure 2D). Quantification of 11–16 organoids from two independent batches showed FOXA2 expression across all cell lines, with the following proportions: HC6#10, 80.2 %; NC01#7, 72.2 %; NC01#14, 80.2 %; PD01#16, 67.3 %; PD01#40, 56.1 %; HPS4264, 59.9 %; and HPS4265, 71.6 %. As FOXA2 immunostaining was quantified in two independent experiments, no comparative statistical analysis or calculation of standard error was performed.

### 3.3 Characterization of dopaminergic progenitors in midbrain organoids

**Figure 3:**
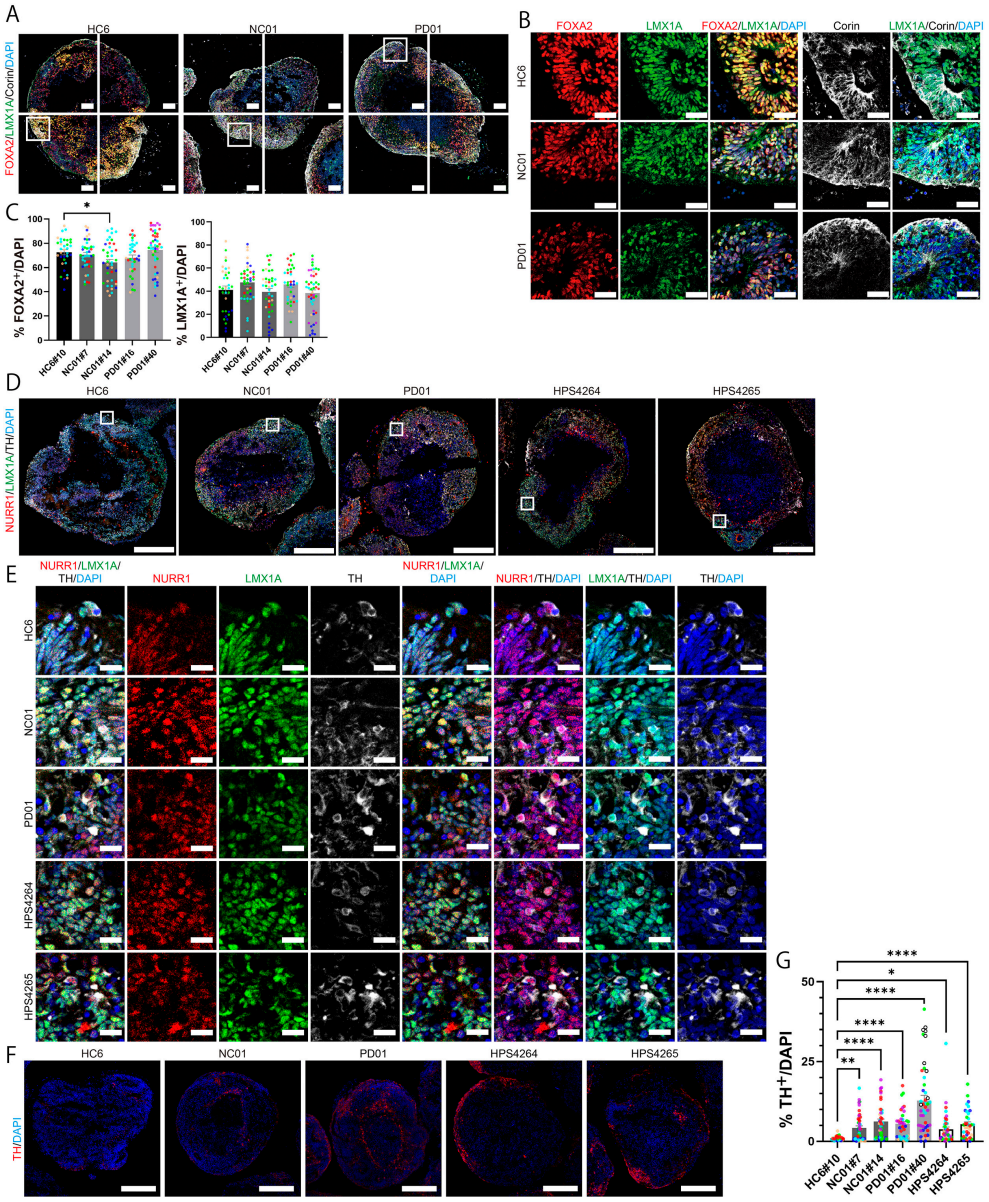
Differentiation of dopaminergic neurons in midbrain organoids. **(A)** Immunostaining of organoids derived from HC6, NC01, and PD01 iPSC lines with FOXA2 (red), LMX1A (green), and Corin (white). Scale bars, 100 μm. **(B)** Representative high-magnification images from each iPSC line are shown. Scale bars, 40 μm. **(C)** Percentage of FOXA2^+^ and LMX1A^+^ cells in organoids on day 28. HC6#10: 5 batches, 5–8 organoids each, *n* = 34; NC01#7: 6 batches, 5–8 organoids each, *n* = 41; NC01#14: 6 batches, 5–8 organoids each, *n* = 40; PD01#16: 6 batches, 3–8 organoids each, *n* = 35; PD01#40: 7 batches, 3–9 organoids each, *n* = 47. Each dot represents an individual organoid, and colors indicate independent differentiation batches within each clone. **(D)** Immunostaining of organoids derived from HC6, NC01, PD01, HPS4264, and HPS4265 with NURR1 (red), LMX1A (green), and TH (white). Scale bars, 500 μm. **(E)** Magnified images of the inset in **(D)**. Scale bars, 40 μm. **(F)** Immunostaining for TH (red) in organoids on day 28. Scale bars, 500 μm. **(G)** Percentage of TH^+^ cells in the organoids on day 28. HC6#10: 5 batches, 4–8 organoids each, *n* = 31; NC01#7: 6 batches, 5–8 organoids each, *n* = 40; NC01#14: 6 batches, 5–8 organoids each, *n* = 40; PD01#16: 6 batches, 3–8 organoids each, *n* = 35; PD01#40: 7 batches, 2–9 organoids each, *n* = 46; HPS4264: 6 batches, 6–8 organoids each, *n* = 45; HPS4265: 5 batches, 6–8 organoids each, *n* = 46. Each dot represents an individual organoid, and colors indicate independent differentiation batches within each clone. Statistical significance: *p < 0.05, **p < 0.01, ****p < 0.0001.

During early development, LMX1A is expressed in the roof plate and the ventral midline region where midbrain dopaminergic neurons emerged, as previously reported (Andersson et al., 2006; Ono et al., 2007). Corin, a transmembrane domain-containing cell-surface protease, is expressed in the floor plate (Ono et al., 2007; Samata et al., 2016). In the E12.5 mouse embryo, the floor plate of the neural tube exhibits expression of multiple markers associated with midbrain dopaminergic neuron development (Supplementary Figure 1). When iPSC neural organoids were further cultured until day 28, a substantial population of FOXA2^+^ cells was observed (Figures 3A, B). Quantification of FOXA2^+^ and LMX1A^+^ cells was performed on the neuroepithelium (200 μm width) in the peripheral region of a single organoid section, excluding the central regions rich in dead cells (Figure 3C). FOXA2^+^ cells in the organoids were 72.51 ± 2.15 % in HC6#10, 70.42 ± 1.92 % in NC01#7, 64.10 ± 2.56 % in NC01#14, 67.98 ± 2.31 % in PD01#16, and 74.55 ± 2.38 % in PD01#40. Compared with HC6, no significant differences were observed in NC01#7, PD01#16, or PD01#40, whereas NC01#14 showed a significant decrease. LMX1A^+^ cells in the organoids were 41.09 ± 3.47 % in HC6#10, 47.54 ± 2.48 % in NC01#7, 39.43 ± 2.87 % in NC01#14, 45.92 ± 2.55 % in PD01#16, and 38.35 ± 2.73 % in PD01#40 (Figure 3C). No significant difference was observed in LMX1A^+^ cells in organoids derived from other iPSC lines compared to HC6#10. Several organoids contained fewer than 30% FOXA2^+^/DAPI^+^ cells (one case in NC01#7, six cases in PD01#16, and four cases in PD01#40). Based on previous reports (Tieng et al., 2014; Jo et al., 2016), where FOXA2^+^/DAPI^+^ ratios below 30% are uncommon, organoids with fewer than 30% FOXA2^+^/DAPI^+^ cells were deemed to exhibit insufficient midbrain differentiation and were excluded from the analysis. Approximately 50% of FOXA2^+^ cells in the neuroepithelium co-expressed LMX1A (Figures 3A, B), indicating that the organoids differentiated into the midbrain floor plate region. Furthermore, the expression of Corin in LMX1A^+^ cells suggests that these cells are differentiating into dopaminergic progenitors in the ventral midbrain by day 28 in culture.

*NURR1* is a transcription factor selectively expressed in midbrain dopaminergic neurons and is involved in regulating their differentiation. We characterized dopaminergic progenitors in iPSC-derived midbrain organoids using immunostaining for NURR1 and tyrosine hydroxylase (TH). On day 28, NURR1 and TH were expressed in the organoids (Figure 3D). NURR1^+^/LMX1A^+^/TH^+^ cells appeared in the neuroepithelium of all organoids, regardless of whether they were control- or patient-derived (Figure 3E). TH^+^ cells in the organoids were 0.94 ± 0.11 % in HC6, 4.18 ± 0.62 % in NC01#7, 6.13 ± 0.84 % in NC01#14, 5.49 ± 0.74 % in PD01#16, 12.76 ± 1.61 % in PD01#40, 3.72 ± 0.75 % in HPS4264, and 5.32 ± 0.72 % in HPS4265 (Figures 3F, G). Compared to HC6#10, a significant increase in TH^+^ cells was noted in the organoids derived from NC01, PD01, and *PRKN* mutant iPSCs.

### 3.4 Differentiation of midbrain dopaminergic neurons is more developed in PD iPSC-derived organoids on day 56

**Figure 4:**
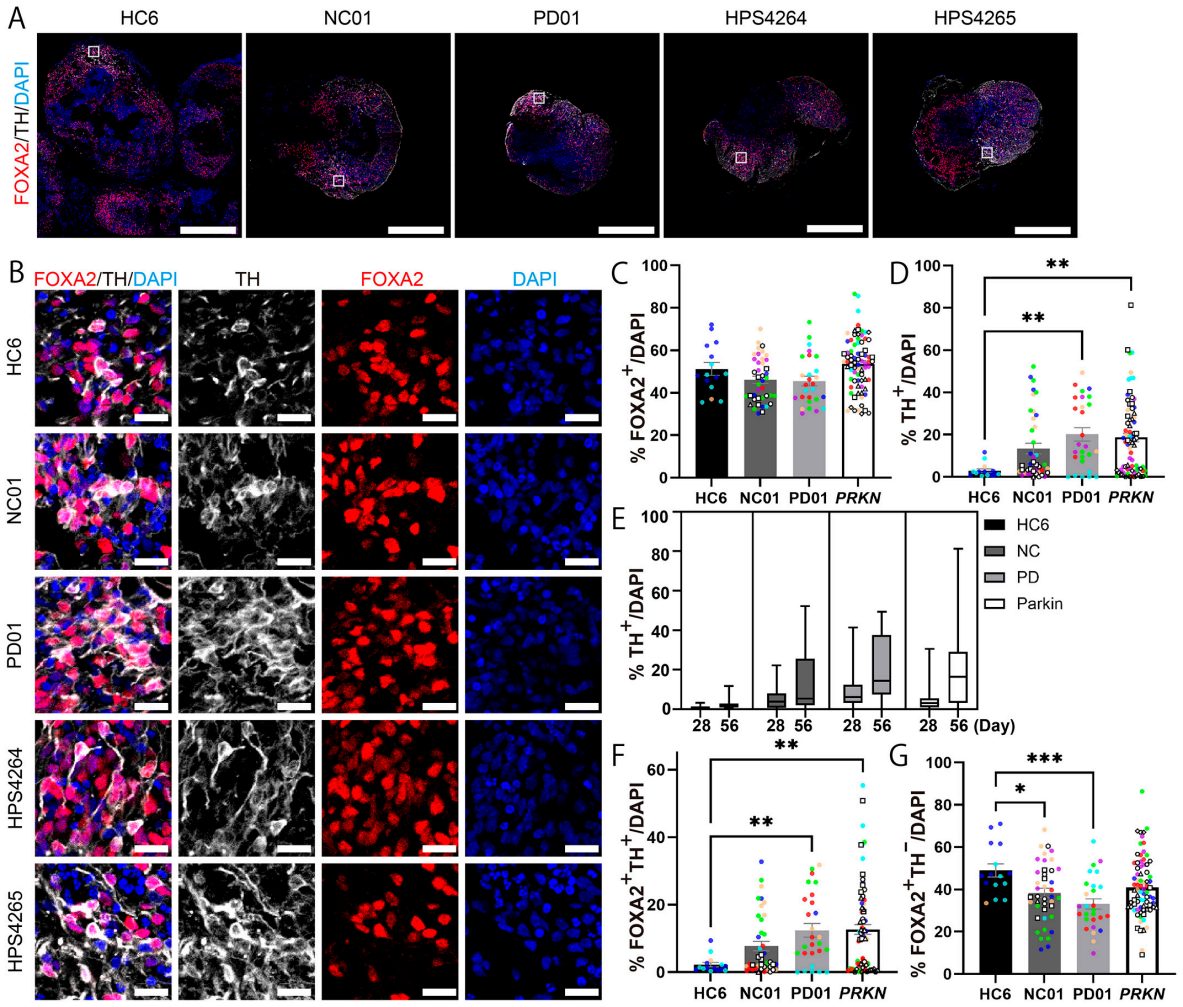
Quantitative analysis of midbrain dopaminergic neurons and progenitors in organoids on day 56. **(A)** Immunostaining of organoids derived from HC6, NC01, PD01, HPS4264, and HPS4265 for FOXA2 (red), TH (white), and DAPI (blue). Scale bars, 500 μm. **(B)** Magnified images of the insets in **(A)**. Scale bars, 40 μm. Percentage of **(C)** FOXA2^+^ cells, **(D)** TH^+^ cells, **(F)** FOXA2^+^/TH^+^cells and **(G)** FOXA2^+^/TH^−^ cells in organoids on day 56. HC6#10: 3 batches, 1–8 organoids each, n = 15; NC01#7 and NC01#14: 4 and 5 batches, 1–8 organoids each, n = 41; PD01#16 and PD01#40: 5 and 1 batches, 1–7 organoids each, n = 27; HPS4264 and HPS4265: 5 and 6 batches, 5–8 organoids each, n = 78. Each dot represents an individual organoid, and colors and shapes indicate independent differentiation batches within each clone. **(E)** Comparison of the percentage of TH^+^ cells in organoids at days 28 and 56. Statistical significance: *p < 0.05, **p < 0.01, ***p < 0.001.

Accelerated differentiation toward midbrain dopaminergic neurons may reduce the relative abundance of dopaminergic progenitors, potentially resulting in fewer mature neurons at later stages. To evaluate temporal changes, immunostaining for TH and FOXA2 was performed on midbrain organoids on day 56 (Figures 4A–G). No significant difference was observed in FOXA2^+^ cells in the organoids derived from the NC01, PD01, and *PRKN* iPSC lines compared to those derived from the HC6 line: 51.24 ± 3.03 % in HC6 organoids, 46.12 ± 1.64 % in NC01 (NC01#7 and NC01#14), 45.52 ± 2.24 % in PD01 (PD01#16 and PD01#40), and 53.74 ± 1.42 % in the *PRKN* (HPS4264 and HPS4265) organoids (Figure 4C). In contrast, TH^+^ cells in the PD01- and *PRKN*-derived organoids were markedly increased compared to HC6 organoids: 2.95 ± 0.83 % in HC6, 13.47 ± 2.46 % in NC01 (NC01#7 and NC01#14), 20.19 ± 3.13 % in PD01 (PD01#16 and PD01#40), and 18.67 ± 2.00 % in *PRKN* (HPS4264 and HPS4265) organoids (Figure 4D). On day 56, the number of TH^+^ cells increased in all iPSC lines compared to day 28 (Figure 4E). The percentage of cells co-expressing FOXA2 and TH, representing midbrain dopaminergic neurons, increased significantly compared to HC6 in organoids derived from the PD01 and *PRKN* cell lines: 2.24 ± 0.64 % in HC6, 7.74 ± 1.40 % in NC01 (NC01#7 and NC01#14), 12.41 ± 2.01 % in PD01 (PD01#16 and PD01#40), and 12.67 ± 1.45 % in *PRKN* (HPS4264 and HPS4265) (Figure 4F). FOXA2 and TH double-positive cells differentiate into midbrain dopaminergic neurons derived from the floor plate. By contrast, FOXA2^+^/TH^−^ cells are presumed to remain as midbrain dopaminergic progenitors. The percentage of FOXA2^+^/TH^−^ cells decreased significantly compared to HC6 in organoids derived from NC01 and PD01: 49.00 ± 3.08 % in HC6, 38.38 ± 2.19 % in NC01, 33.11 ± 2.42% in PD01, and 41.06 ± 1.57 % in *PRKN* (Figure 4G).

## 4 Discussion

In this study, we first established patient-derived iPSCs and confirmed their pluripotency and the retention of disease-associated genetic mutations (Figure 1). We developed a protocol to differentiate these iPSCs into midbrain organoids with neuroepithelial structures corresponding to the floor plate region of the neural tube (Figure 2), validating their suitability as a disease model. The midbrain organoids generated using this protocol formed neuroepithelial structures by day 14 and differentiated into midbrain dopaminergic progenitors by day 28, as confirmed via immunostaining for FOXA2, LMX1A, and Corin (Figures 3A–C), thus appropriately recapitulating the process of midbrain dopaminergic neurogenesis. The healthy control iPSC line HC6 was matched to the other iPSC lines with regard to race, gender and age. HC6 has been used in various studies of neurological disorders; spinal motor neurons for amyotrophic lateral sclerosis (ALS) (Kume et al., 2023) and Purkinje cells for spinocerebellar degeneration (SCD) (Ishida et al., 2016). In present study, HC6 iPSCs showed a capacity to differentiate into TH^+^ dopaminergic neurons in midbrain organoids that was almost equivalent to that reported previously (Ogura et al., 2013; Abbott et al., 2023). It is important to note that, to minimize variability between experiments, midbrain organoids were generated from all iPSC lines simultaneously. The results are consistent with previous reports and support the reliability of the model (Jo et al., 2016; Smits et al., 2019).

At this stage, TH-positive cells were observed in all cell lines (Figures 3D, E), with a tendency for higher proportions in NC, PD, and *PRKN* lines compared with healthy controls (Figures 3F, G). By day 56, FOXA2^+^/TH^+^ dopaminergic neurons were increased in PD and *PRKN* lines relative to controls (Figures 4A–F), whereas FOXA2^+^/TH^−^ dopaminergic progenitors were significantly reduced in the NC and PD cell lines, with the *PRKN* line showing a similar tendency toward reduction (Figure 4G). While our data do not directly demonstrate progenitor exhaustion or late-stage neuronal loss, the observed shift in the FOXA2^+^/TH^−^ population leads us to hypothesize that accelerated dopaminergic differentiation might eventually deplete the progenitor pool, potentially leading to a reduced number of dopaminergic neurons at birth.

Compared with healthy control, early-onset PD and *PRKN* lines exhibited enhanced differentiation into midbrain dopaminergic neurons, accompanied by a tendency toward reduced progenitor populations. Maintaining a sufficient pool of progenitors and balancing proliferation with differentiation during neurogenesis is essential for generating an adequate number of mature neurons (Petersen et al., 2002; Chou and O'Leary, 2013). Moreover, enhanced maturation can disrupt proper temporal formation of neural circuits, ultimately reducing the number of neurons. The se findings suggest that in patients with 22q11.2 DS or PRKN mutations who develop early-onset PD, enhanced differentiation into midbrain dopaminergic neurons may result in a reduced number of dopaminergic neurons from birth. These results imply that the pathophysiology of PD is not merely age-related neurodegeneration but may also involve developmental quantitative abnormalities in midbrain dopaminergic neurons.

In our comparative analysis, we noted substantial differences between the 22q11.2 DS-PD (PD01) line and the PRKN mutant lines regarding cellular morphology and the proportions of TH^+^ and FOXA2^+^ populations during differentiation. These differences are highly likely attributable to the distinct molecular pathophysiology of each genetic defect. The PRKN mutation primarily triggers downstream mitochondrial distress and impaired mitophagy, which exerts a relatively subtle influence on early neural lineage specification. On the other hand, the 22q11.2 deletion in the PD01 line encompasses crucial neurodevelopmental drivers, including DiGeorge syndrome critical region gene 8 (DGCR8) and RAN binding protein 1 (RANBP1), which directly orchestrate cell-cycle exit and neuronal pacing. Consequently, the hemizygosity of the 22q11.2 locus leads to a much more pronounced, accelerated dopaminergic differentiation phenotype compared to the PRKN lines. This divergence underscores that while both lines model Parkinson's disease, the early-stage neurodevelopmental aberrations are characteristically driven by the specific genetic architecture of the 22q11.2 deletion.

Our findings highlight how key genes within the 22q11.2 deletion region—namely DGCR8, RANBP1, and catechol-O-methyltransferase (COMT) (Mok et al., 2016)—impact the cell cycle, proliferation, and differentiation of neural stem/progenitor cells (NSPCs). The loss of DGCR8 impairs miRNA-mediated gene regulation (Chen et al., 2012), while RANBP1 deficiency disrupts mitotic progression and nucleocytoplasmic transport (Paronett et al., 2015), both contributing to a decreased progenitor pool. Coupled with altered catecholamine signaling via COMT dysfunction, these molecular deficits collectively push NSPCs out of the cell cycle. This shifts their fate toward accelerated, premature differentiation. Consequently, the synergy of these microdeletion targets underlies the neurodevelopmental phenotypes observed in our model.

Furthermore, NC lines derived from unaffected family members, who did not develop PD even at advanced age, exhibited a tendency toward enhanced maturation of midbrain dopaminergic neurons. These cell lines lack the 22q11.2 deletion and *PRKN* mutations. This observation suggests that, in addition to enhanced differentiation-related reduction of dopaminergic neurons, other factors such as modifier genes or unidentified genetic predispositions may contribute to the development of early-onset PD, indicating its multifactorial etiology.

This study has certain several limitations. Firstly, we have demonstrated that the differentiation of dopaminergic neurons is enhanced in midbrain organoids derived from 22q11.2 DS and *PRKN* mutant iPSCs from a single patient. However, we have not confirmed whether this is a characteristic specific to early-onset PD. In the future, it will be necessary to investigate whether this enhanced differentiation is specific to early-onset PD by using iPSCs derived from late-onset PD. Secondly, although we were able to demonstrate quantitative abnormalities in dopaminergic neurons, qualitative defects were not sufficiently addressed. To elucidate these aspects, analyses such as electrophysiological recordings to assess firing properties and autoreceptor responses, measurements of dopamine release and reuptake, as well as evaluation of A9 subtype-specific markers and mitochondrial function, would be informative. These approaches could clarify neuronal maturation and functional vulnerability. Furthermore, to advance the findings of this study, it is necessary to investigate the involvement of unidentified genetic predispositions and modifier factors. Approaches such as whole-genome sequencing, pedigree analysis, and polygenic risk score assessment, combined with single-cell RNA sequencing and epigenomic profiling of organoids, would be valuable. In addition, introducing or correcting candidate genes using CRISPR followed by functional assays could directly test the causal roles of these factors.

## 5 Conclusion

This study demonstrates that enhanced differentiation of midbrain dopaminergic progenitors represents an atypical development in models of early-onset PD. These findings suggest that PD pathophysiology may not be limited to age-related neurodegeneration but could involve neurodevelopmental defects established during embryogenesis. Moreover, the number of dopaminergic neurons may already be reduced at birth, highlighting the need for strategies aimed at early diagnostic biomarkers and preventive interventions.

## Data Availability

The original contributions presented in the study are included in the article/Supplementary material, further inquiries can be directed to the corresponding author.
