# Supplementary material for: Neurogenesis defects in iPSC-derived midbrain organoids of early-onset Parkinson's disease with 22q11.2 deletion syndrome
Source: Front Cell Neurosci. 2026 Jul 31;20:1906023. doi: 10.3389/fncel.2026.1906023 (PMC13472798; doi:10.3389/fncel.2026.1906023)
Supplement: Supplementary file 1 [file Data_Sheet_1.pdf]

donor information	control 1	control 2		patient 1		patient 2	
iPSC line	HC6#10	NC01#7	NC01#14	PD01#16	PD01#40	HPS4264	HPS4265
gender	female	female		male		female	
race	Japanese	Japanese		Japanese		Japanese	
age of biopsy	49	70s		48		54	
diagnosis	n/a	n/a		PD		PD	
variant	n/a	n/a		22q11.2DS		*PRKN* c.1341G>A (p.W447Ter)	
age of onset	n/a	n/a		39		unknown	
somatic cell for iPSC	PBMCs	PBMCs		PBMCs		PBMCs	
vector for reprograming	episomal	episomal		episomal		episomal	
genes for programing	Oct3/4, Sox2, Klf4, L-Myc, Lin28, EBNA1, p53DD	Oct3/4, Sox2, Klf4, L-Myc, Lin28, EBNA1, p53DD		Oct3/4, Sox2, Klf4, L-Myc, Lin28, EBNA1, p53DD		Oct3/4, Sox2, Klf4, L-Myc, Lin28, EBNA1, p53DD	

Supplementary Table 1. Information on iPSCs used in this study.
Information for iPSCs derived from a healthy donor, a PD patient with 22q11.2 DS (PD01#16 and PD01#40), an unaffected blood relative (NC01#7 and NC01#14), and a PD patient with *PRKN* mutations (HPS4264 and HPS4265).
PBMC: peripheral blood mononuclear cell, n/a: not applicable, PD: Parkinson’s disease.
