# Supplementary material for: Neurogenesis defects in iPSC-derived midbrain organoids of early-onset Parkinson's disease with 22q11.2 deletion syndrome
Source: Front Cell Neurosci. 2026 Jul 31;20:1906023. doi: 10.3389/fncel.2026.1906023 (PMC13472798; doi:10.3389/fncel.2026.1906023)
Supplement: Supplementary file 2 [file Data_Sheet_2.pdf]

Supplementary Table 2 (related to Figures 2-4)
List of primary and secondary antibodies

Antibody	Manufacturer	Cat No.	Host	Dilution
Corin	R&D systems	MAB2209	rat	500
FOXA2	Cell Signaling	8186S	rabbit	400
Laminin	abcam	ab11575	rabbit	1,000
Lmx1a	Custom	MS1469-72	guinea pig	10,000
Nestin	Atlas	AMAb90556	mouse	1,000
Nurr1	R&D systems	AF2156	goat	200
Phospho-Histone H3	Cell Signaling	9706	mouse	1,000
PKC zeta	Santa Cruz Biotechnology	sc-216	mouse	100
SOX2	Santa Cruz Biotechnology	sc-17320	goat	300
Tyrosine hydroxylase	Millipore	AB1542	sheep	200
Tyrosine hydroxylase	Millipore	AB152	rabbit	500
Cy3 anti-rabbit IgG	Jackson Immuno Research	711-165-152	donkey	400
Cy3 anti-mouse IgG	Jackson Immuno Research	715-165-151	donkey	400
Cy5 anti-mouse IgG	Jackson Immuno Research	715-175-151	donkey	400
Alexa Fluor647 anti-mouse IgG	Jackson Immuno Research	715-605-151	donkey	500
Alexa Fluorn 488 anti-rabbit IgG	Thermo Fisher Scientific	A11034	goat	1,000
Alexa Fluorn 568 anti-mouse IgG	Thermo Fisher Scientific	A11004	goat	1,000
Alexa Fluor488 anti-mouse IgG	Jackson Immuno Research	715-545-151	donkey	500
Alexa Fluor488 anti-rabbit IgG	Jackson Immuno Research	711-545-152	donkey	500
Alexa Fluor488 anti-goat IgG	Jackson Immuno Research	705-545-147	donkey	500
Cy3 anti-rabbit IgG	Jackson Immuno Research	711-165-152	donkey	400
Cy3 anti-mouse IgG	Jackson Immuno Research	715-165-151	donkey	400
Cy5 anti-mouse IgG	Jackson Immuno Research	715-175-151	donkey	400
Alexa Fluor647 anti-mouse IgG	Jackson Immuno Research	715-605-151	donkey	500

Supplementary Table 2
List of primary and secondary antibodies used in this study.
