# Supplementary material for: Neurogenesis defects in iPSC-derived midbrain organoids of early-onset Parkinson's disease with 22q11.2 deletion syndrome
Source: Front Cell Neurosci. 2026 Jul 31;20:1906023. doi: 10.3389/fncel.2026.1906023 (PMC13472798; doi:10.3389/fncel.2026.1906023)
Supplement: Supplementary file 3 [file Data_Sheet_3.pdf]

A. Number of organoids from each iPSC line used for the quantitative analysys of FOXA2 on day 14 (related to Figure 2)

iPSC line	Antibodies used in IHC	Experimental replicates	Total number of organoids	Each number of organoids per batch
HC6#10	FOXA2	2	12	7, 5
NC01#7	FOXA2	2	12	6, 6
NC01#14	FOXA2	2	14	6, 8
PD01#16	FOXA2	2	14	6, 8
PD01#40	FOXA2	2	15	4, 7
HPS4264	FOXA2	2	14	6, 8
HPS4265	FOXA2	2	16	8, 8

B. Number of organoids from each iPSC line used for the quantitative analysys of FOXA2/LMX1A and TH on day 28 (related to Figure 3)

iPSC line	Antibodies used in IHC	Experimental replicates	Total number of organoids	Each number of organoids per batch
HC6#10	FOXA2/LMX1A	5	34	8, 6, 5, 7, 8
NC01#7	FOXA2/LMX1A	6	41	5, 7, 8, 7, 8, 6
NC01#14	FOXA2/LMX1A	6	40	7, 5, 7, 8, 8, 5
PD01#16	FOXA2/LMX1A	6	35	3, 4, 8, 5, 8, 7
PD01#40	FOXA2/LMX1A	7	47	3, 8, 7, 5, 7, 9, 8

HC6#10	TH	5	31	8, 6, 5, 4, 8
NC01#7	TH	6	40	5, 7, 8, 7, 7, 6
NC01#14	TH	6	40	7, 5, 7, 8, 8, 5
PD01#16	TH	6	35	3, 4, 8, 5, 8, 7
PD01#40	TH	7	46	2, 8, 7, 5, 7, 9, 8
HPS4264	TH	6	45	8, 6, 8, 8, 8, 7
HPS4265	TH	5	46	6, 6, 8, 8, 8

C. Number of organoids from each iPSC line used for the quantitative analysys of FOXA2/TH on day 56 (related to Figure 4)

iPSC line	Antibodies used in IHC	Experimental replicates	Total number of organoids	Each number of organoids per batch
HC6#10	FOXA2/TH	3	15	6, 1, 8
NC01#7	FOXA2/TH	4	21	7, 5, 1, 8
NC01#14	FOXA2/TH	5	20	6, 6, 1, 1, 6
PD01#16	FOXA2/TH	5	23	1, 6, 6, 3, 7
PD01#40	FOXA2/TH	1	4	4
HPS4264	FOXA2/TH	5	34	5, 8, 8, 7, 6
HPS4265	FOXA2/TH	6	44	8, 7, 8, 5, 8, 8

Supplementary Table 3. Summary of independent differentiation batches and organoids analyzed.
